# Very Similar Spacing-Effect Patterns in Very Different Learning/Practice Domains

**DOI:** 10.1371/journal.pone.0090656

**Published:** 2014-03-07

**Authors:** Jürgen Kornmeier, Manfred Spitzer, Zrinka Sosic-Vasic

**Affiliations:** 1 Institute for Frontier Areas of Psychology and Mental Health, Freiburg, Germany; 2 Eye Center, Albert-Ludwigs-University of Freiburg, Freiburg, Germany; 3 Department of Psychiatry and Psychotherapy, University Clinic of Ulm, Ulm, Germany; 4 Transfer Center for Neuroscience and Learning, University of Ulm, Ulm, Germany; University of Sydney, Australia

## Abstract

Temporally distributed (“spaced”) learning can be twice as efficient as massed learning. This “spacing effect” occurs with a broad spectrum of learning materials, with humans of different ages, with non-human vertebrates and also invertebrates. This indicates, that very basic learning mechanisms are at work (“generality”). Although most studies so far focused on very narrow spacing interval ranges, there is some evidence for a non-monotonic behavior of this “spacing effect” (“nonlinearity”) with optimal spacing intervals at different time scales. In the current study we focused both the nonlinearity aspect by using a broad range of spacing intervals and the generality aspect by using very different learning/practice domains: Participants learned German-Japanese word pairs and performed visual acuity tests. For each of six groups we used a different spacing interval between learning/practice units from 7 min to 24 h in logarithmic steps. Memory retention was studied in three consecutive final tests, one, seven and 28 days after the final learning unit. For both the vocabulary learning and visual acuity performance we found a highly significant effect of the factor spacing interval on the final test performance. In the 12 h-spacing-group about 85% of the learned words stayed in memory and nearly all of the visual acuity gain was preserved. In the 24 h-spacing-group, in contrast, only about 33% of the learned words were retained and the visual acuity gain dropped to zero. The very similar patterns of results from the two very different learning/practice domains point to similar underlying mechanisms. Further, our results indicate spacing in the range of 12 hours as optimal. A second peak may be around a spacing interval of 20 min but here the data are less clear. We discuss relations between our results and basic learning at the neuronal level.

## Introduction

How can we efficiently optimize learning? The answer to this question is of high practical relevance for all of us but also of theoretical interest for the basic understanding of learning and memory. One answer lies in the timing of learning units: Temporally distributed learning units over a longer time period are more efficient than massed learning temporally close to the test.

### The spacing effect

This phenomenon is called the “spacing effect” (or alternatively “lag effect” or “distributed practice effect”) and it has been studied scientifically as early as in the 19^th^ century by Herrmann Ebbinghaus [1]. Spaced learning can be more than twice as efficient than massed learning if the appropriate spacing duration is used [2]. Spacing effects occur with a broad spectrum of learning materials, like sense and nonsense syllables, words, word pairs (e.g. vocabulary learning), grammatical rules, history facts, pictures, arithmetic rules, scientific terms and concepts (e.g. in mathematics), and even with motor skills, e.g. [3], [4]. Spacing effects have been found with multimodal (audio-visual) stimulus material, e.g. [5], both with intentional and incidental learning, e.g. [6], [7], and for a broad spectrum of ages from 4-year-old children [8], [9] up to 76 year-old seniors, e.g. [10], [11]. Spacing effects have been found in animals such as rodents, e.g. [12], the sea slug Aplysia, e.g. [13], and even within the fruit fly Drosophila, e.g. [14].

This generality of the spacing effect indicates that the underlying learning mechanisms are very basic.

### The testing effect

Tests are commonly regarded as tools to control the learning success and too many tests seem to waste important time necessary for learning, e.g. [15]. The testing effect, however, demonstrates that testing can be much more efficient for learning than simple repetitions and that appropriately combining tests and repetitions of learning material can significantly improve performance and/or reduce the necessary time for learning, e.g. [16], [17]. Moreover spaced practice (a combination of repetitions and tests) is better than massed practice, e.g. [3], [18], for a variety of learning materials/situations, e.g. [3].

### Focus of the current study

In the current study we mainly focused at two aspects:

#### 1. Extending the within-study range of spacing and retention intervals

Most studies so far focused on very narrow spacing interval ranges and only one or two “retention intervals”, i.e. the temporal distance between the last practice unit and the final test, e.g. [2]. And thus only a small number of studies report about a non-monotonic pattern of the spacing effect showing increased learning performance with optimal spacing intervals and decreasing performance with shorter and longer spacing intervals, e.g. [2], [3], [19]. Further, the optimal spacing interval duration seems to increase with the retention interval duration [4], [19]. Modeling the dependence of learning performance on spacing and retention intervals as a three-dimensional space (“Spacing-Retention-Performance Space”  =  “SRP-Space”), we have recently suggested that this SRP-Space contains more than one peak, i.e. more than one optimal combination of spacing and retention intervals [20].

In the current study we used an extended range of spacing intervals from 7 min (minimum) to 24 h (maximum) in six logarithmic steps within one study. Further, we applied three final tests at retention intervals of 1 day, 7 days and 28 days. Within this extended subspace we looked for the number of within-study peaks.

#### 2. Testing for generality of the spacing effect

**Figure 1:**
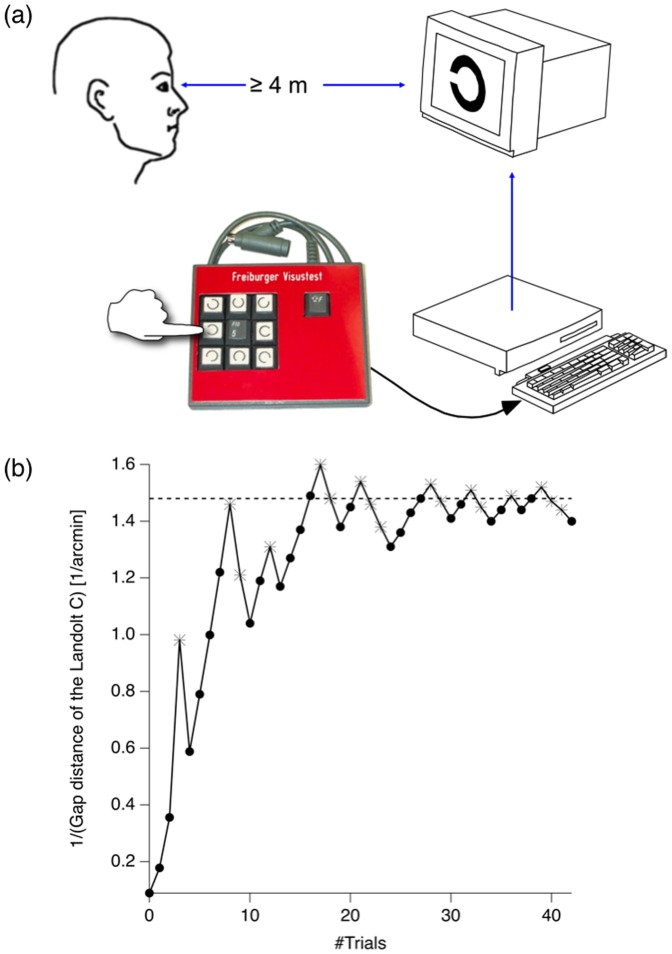
Visual Acuity Test. (a) Typical setup of the visual acuity test. Participants indicate by key the perceived or guessed (forced choice) position of the Landolt gap. (b) Representative example of a FrACT (Bach & Kommerell 1998) visual acuity test run of one participant. Visual acuity is estimated with an adaptive algorithm (Best PEST Algorithm, Lieberman & Pentland 1982) starting with large optotypes (Landolt Cs) and decreasing step sizes depending on the correctness of participant's response. The optotype size converges on a threshold value (1.45) that is used as the participant's visual acuity. (•) correct responses; (*) incorrect responses.

The generality of the spacing effect across learning materials, learning conditions, learning contexts, age and species, strongly indicates that the underlying mechanisms are very basic for learning and memory formation. In the current study we tested the generality within one study with very different learning/practice tasks. In one experiment participants performed a classical paired associated learning task (i.e. vocabulary lists), where we combined repeated presentations and tests with feedback. In the second experiment the same participants performed visual acuity tests temporally close to the vocabulary-learning task (see Fig. 1). It is known that visual acuity can increase with practice and Heinrich et al. [21] have recently shown that the spacing between practice units is relevant. Visual acuity performance and vocabulary learning are very different tasks and most probably recruit very different brain areas. Similar optimal and suboptimal spacing interval durations and similar dependencies of spacing and retention intervals in these very different tasks would be strong within-study evidence for generalized and thus basic learning mechanisms.

## Methods

### Participants

120 healthy participants recruited via advertisements at the local university took part. The data of some participants could not enter the analysis due to misses or delays of one or more experimental sessions. All participants whose data entered the analysis were right-handed as assessed by the Edinburgh Handedness Inventory [22] and had a mean age of 23.3 years (vocabulary experiment) and 23.6 (visual acuity tests). Further details are listed in Table 1. Depending on the assigned experimental condition and the participation time needed to be invested within one group, all participants were paid between 20 Euros and 100 Euros for their participation. In addition, to keep up motivation for this rather time-consuming experiment, a voucher was awarded by random draw among those participants that did not miss any of the experimental sessions.

**Table 1:** Participants' age and gender (S1-S6: spacing conditions).

	Experiment	Age Mean	Age SD	#Females	#Males
**S1**	Vocuabulary	24.6	6.7	15	5
	Visus	24.9	7.4	11	5
**S2**	Vocuabulary	25	9.8	14	3
	Visus	26	11.4	9	3
**S3**	Vocabulary	23.8	8.5	11	5
	Visus	24.4	7.7	15	5
**S4**	Vocuabulary	22.4	5.4	13	5
	Visus	23.2	5.7	12	5
**S5**	Vocabulary	22.2	1.8	11	7
	Visus	22.3	1.8	11	7
**S6**	Vocuabulary	21.6	10.8	8	10
	Visus	21.9	11	7	10

The study was conducted with healthy normal participants and contained no invasive measurement. During the experiment, participants simply perceived icons or read words from a computer screen and/or typed words with a computer keyboard. No experimental block lasted longer than 15 minutes and participants were allowed to pause or stop the experiment at any time. The study contained no danger to the participants' health at any moment. We informed the participants at the beginning about the experimental details and the aim to study effects of distributed learning. Written informed consent was obtained from each individual participant. The study was conducted in full accordance with the Declaration of Helsinki. Given these experimental conditions we regarded a statement from the local ethics committee as not necessary.

### Experimental Paradigms

#### Vocabulary Test

**Figure 2:**
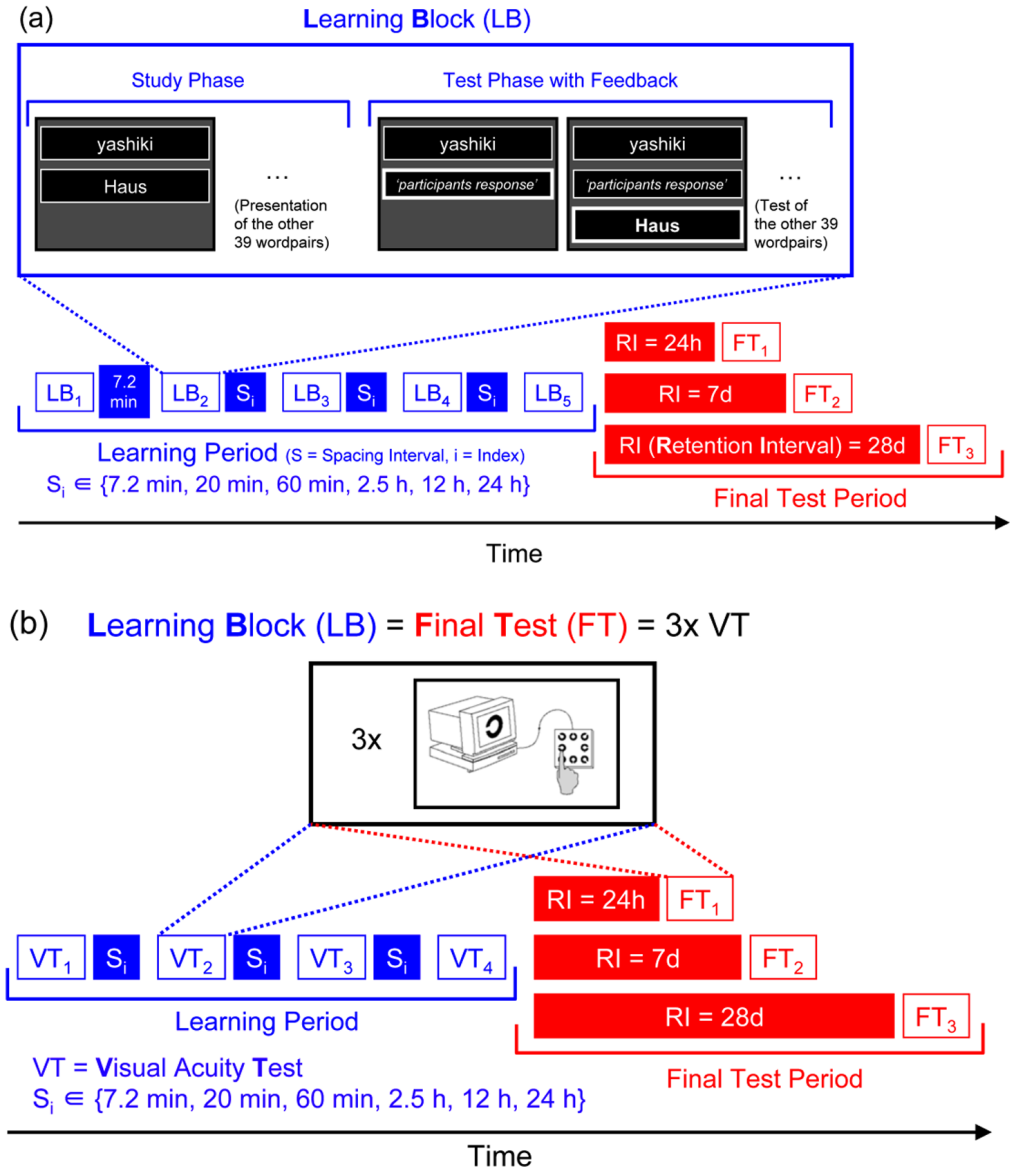
Experimental Paradigms. (a) Vocabulary Experiment: In the study phase of each Learning Block (LB, blue-framed squares) participants viewed and had to learn 40 Japanese-German word pairs. In a subsequent cued-recall test (test phase) the Japanese words were presented and the German translations had to be recalled. A learning block was finished by a 1-s presentation of the correct word as feedback. Initially all participants performed two Learning Blocks separated by 7.2 min. The subsequent three learning blocks were spaced by a condition-specific time S_i_. (b) Visual Acuity Experiment: In the visual acuity experiment participants had to indicate the gap position of a size-varying Landolt-C by key-press. In this experiment, each of the four “Learning Blocks” (blue-framed squares) and each of the three final tests (red-framed squares) consisted of three consecutive visual acuity tests. As in the vocabulary experiment the 6 different experimental conditions differed in the spacing interval durations S_i_ between Learning Blocks.

All participants underwent a verbal paired-associate learning task, which included Japanese-German vocabulary learning: The Japanese words were taken for the sake of phonological novelty compared with most European languages. None of the participants were acquainted with Japanese language or Japanese culture as assessed by interviews prior testing. Each learning block (LB) consisted of a *study phase* and a *test phase* (Fig. 2a).

During the *study phase* participants studied 40 successively presented Japanese-German vocabulary pairs comprising common day-to-day words (e.g. Japanese: yashiki, German: Haus). The word pairs were presented one below the other in the middle of the computer screen for 5 seconds each via Igor Pro 6.22 software (wavemetrics). For each word pair the Japanese words were correct transliterations in Roman alphabet of the German words. Participants were instructed to read and remember the word pairs.

The study phase was followed by a *test phase* with a cued-recall test: The 40 Japanese cue words were presented successively and participants had to type-in the German translation during up to 8 seconds presentation time of the Japanese word and to press the return key at the end of the entry. The return key stopped the entry time-window and started the feedback time-window, where the computer program displayed the correct German translation of the Japanese word for 1 second below the entry field (Fig. 2a). In cases when participants did not provide an entry, the computer program stopped the entry time-window after 8 seconds automatically and displayed the feedback.

Participants' responses were recorded by the computer program. The word pair sequences of both the study and test phase were randomized between blocks, conditions and participants.

In order to prevent potential floor effects, the initial block of the vocabulary learning period (i.e. before any spacing interval) was run twice with a spacing of 7.2 min between blocks for all participants. The remaining three learning blocks were executed after condition-specific spacing intervals.

The three final tests (FT) were identical to the test phase from the learning period, i.e. all participants performed a cued-recall test with feedback but did not restudy the word pairs.

In summary, the vocabulary-learning experiment contained five learning blocks, each with a *study phase* and a subsequent cued-recall test in the *test phase*. The first two learning blocks were spaced by 7.2 min for all participants. The remaining three learning blocks were spaced by one out of six condition-specific times.

#### Visual acuity experiment

Visual acuity tests were performed with the Freiburg Visual Acuity Test that has been developed by Bach [23]. The visual acuity is defined as one above the Landolt gap size at the spatial resolution threshold. The visual acuity test presented 50 Landolt Cs with varying size and 8 possible and randomly changing gap positions. Participants indicated by keyboard the recognition of the Landolt gap (Fig. 1a). The Visual Acuity Test starts with very large and thus easily recognizable Landolts. Depending on the correctness of participant's response subsequent Landolt Cs decrease or increase with decreasing step size, i.e. the difference between Landolt sizes get smaller the closer the test gets to the participant's acuity threshold (Fig. 1b, adaptive Best PEST Algorithm [24]). In summary, the visual-acuity experiment contained four visual acuity test blocks, each with three consecutive visual acuity tests. The visual acuity test blocks were spaced by one out of six condition-specific spacing times.

### Study Design

In order to investigate optimal spacing- and testing-maxima in different learning/practice tasks we chose a 6×3 mixed-model-design:

Spacing interval duration was manipulated between subjects resulting in six spacing conditions (7.2 min, 20 min, 60 min, 2.5 h, 12 h and 24 h). Participants were randomly assigned to one of these six conditions. Participants in the conditions with the four shortest spacing intervals (7.2 min, 20 min, 60 min and 2.5 hours) completed all learning blocks within one day. Participants in the 12-h spacing condition underwent the learning blocks over the course of three days, while participants in the 24-h spacing condition underwent the learning blocks over the course of four days.

The retention interval durations (temporal delay between the end of the last learning block and a final test: 24 hours, 7 days, or 28 days) were manipulated within participants.

During each experimental session all participants underwent both, the vocabulary experiment and the visual acuity experiment, described in detail above. In the conditions with spacing interval durations ≥60 min vocabulary blocks and visual acuity blocks were nested, whereas in conditions with spacing intervals <60 min the learning block of one experiment had to be completed before the other started. The experiment started either with the vocabulary or the visual acuity experiment (randomized across participants).

Participants were tested individually in our lab. Prior to the experiment they were told that they would study and recall word pairs across a series of learning and test trials, and in addition, will undergo a series of visual acuity tests. In the two shortest spacing interval conditions, participants were engaged in filler tasks (watching a short sketch sequence on the computer for about 7 minutes) between the learning blocks in order to prevent immediate silent rehearsal (in the case of vocabulary learning). The filler task took place at the start of the spacing interval. In the remaining four longer spacing interval conditions, the filler task was restricted to the first spacing interval, which was 7 min for all conditions (see the *Vocabulary Test* section above).

During the final tests all participants performed three succeeding visual acuity tests and a cued-recall test on each of the 40 previously learned Japanese-German word-pairs without restudying the words. After the last final test (after 28 days) with the end of the experiment, participants were debriefed and thanked for their participation.

### Statistics

For each of the 6 spacing groups we collected data from 20 participants. According to the very demanding schedule of the experiment each participant had to visit the lab between four and seven times. We had to skip data of some subjects due to misses or delays of one or more experimental sessions. The number of participants per spacing group entering the data analyses is listed in Tables 2 and 3. Some of the visual acuity data were lost due to technical problems. This explains occasional differences in the number of participants per experiment in Table 2 and 3.

**Table 2:** Average applied spacing and retention intervals.

Intended Spacing	AVG Space Lerning	AVG Space Test1: 24 [h]	AVG Space Test2: 7 [d]	AVG Space Test3: 28 [d]
**7 min**	11±2.7 min (16/20)	23.8±3.4 h (16/20)	6±0.2 d (15/20)	26.9±0.3 d (5/9)
**20 min**	20.2±2.7 min (12/20)	22.3±3.8 h (12/20)	5.9±0.2 d (12,20)	26.8±0.3 d (4/10)
**60 min**	61.5±6.6 min (20/20)	22.2±4.9 h (20/20)	6±0.2 d (20/20)	27.2±0.8 d (10/10)
**150 min**	150.2±8.4 min (17/20)	18.6±3.5 h (17/20)	6±0.2 d (15/20)	25.9±2.8 d (6/10)
**12 h**	16.4±6.3 h (18/20)	21.4±4.8 h (18/20)	6±0.1 d (18/20)	26.1±1.9 d (4/10)
**24 h**	24.2±3.3 h (17/20)	24.4±2.5 h (17/20)	5.9±0.4 d (15/20)	26.7±0.5 d (7/7)

Average applied spacing intervals (column 2) and retention intervals (columns 3–5) ± SEM per spacing condition for the visual acuity experiment (numbers in brackets: number of participants entering analysis/number of total participants).

**Table 3:** Average applied spacing and retention intervals.

Intended Spacing	AVG Space Lerning [min]	AVG Space Test1 [h]	AVG Space Test2 [d]	AVG Space Test3 [d]
**7 min**	15.1±4 min (20/20)	23.3±4 h (20/20)	6±0.2 d (18/20)	27.7±2.6 d (9/9)
**20 min**	28.4±7.6 min (17/20)	21±2.5 h (17/20)	5.6±1.5 d (17/20)	26.4±1.7 d (8/10)
**60 min**	62.2±7.5 min (16/20)	21.6±5.4 h (15/20)	6.1±0.2 d (16/20)	27.3±0.8 d (9/10)
**150 min**	152.5±8.5 min (18/20)	19.2±3.2 h (18/17)	6±0.2 d (16/15)	26.2±2.4 d (10/10)
**12 h**	16.1±6.4 h (18/20)	22.4±6.7 h (18/20)	6±0.3 d (17/20)	26.7±0.8 d (6/10)
**24 h**	23.7±1 h (18/20)	24.4±2.3 h (18/20)	5.8±0.3 d (15/20)	21±0.2 d (6/7)

Average applied spacing intervals (column 2) and retention intervals (columns 3–5) ± SEM per spacing condition for the vocabulary experiment (numbers in brackets: number of participants entering analysis/number of total participants).

### Pre-processing of the vocabulary data

**Figure 3:**
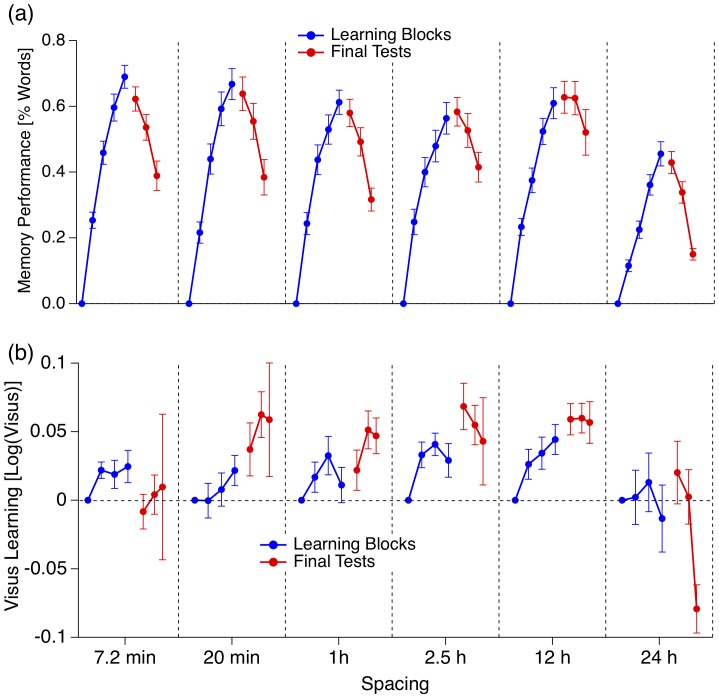
Grand Mean of the test results from the learning period (blue traces) and test results from the final test period (red traces) ± SEM. (a) Results from the vocabulary experiment in % of the total number of presented vocabulary word pairs ( = 40). Maximal performance decreases with spacing interval duration but best long-term retention is found with a spacing interval of 12 h. (b) Results from the visual acuity experiment as the positive logarithm of the maximal visual acuity. Visual acuity is defined as one over the minimal angle of resolution. Again a spacing interval of 12 h produces best long-term retention, although variability is larger compared to (a).

**Figure 4:**
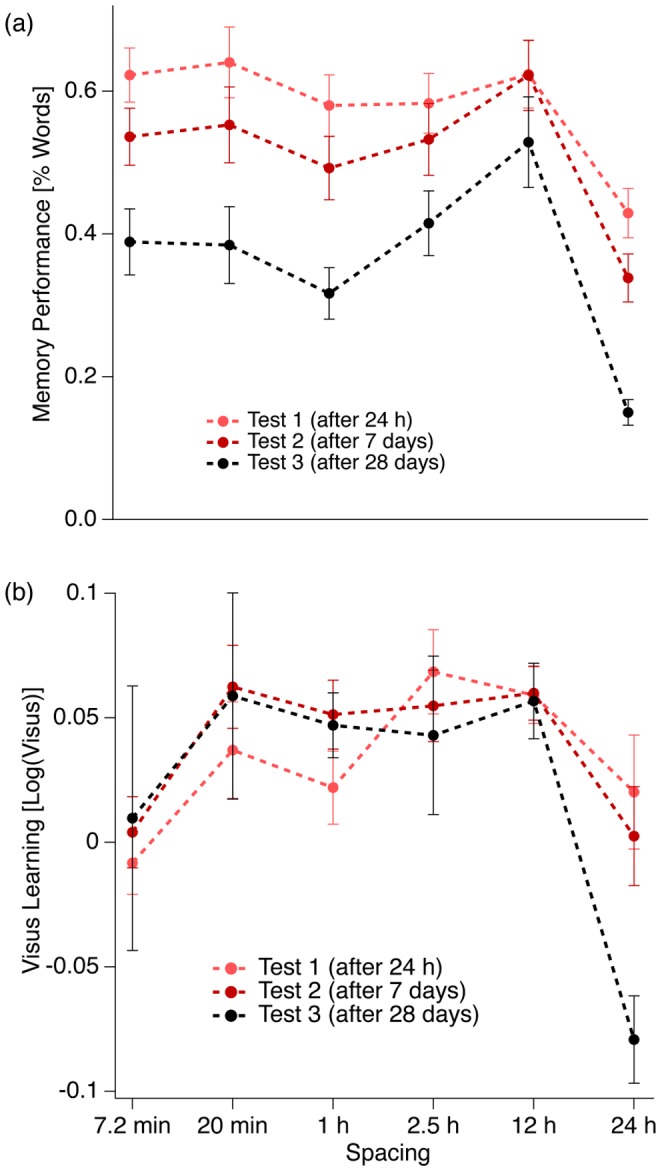
Final Test performance as a function of spacing interval duration (abscissa), separately for each retention interval duration (different traces). (a) Vocabulary experiment: The data are % values of the total number of presented vocabulary word pairs ( = 40), averaged across participants. (b) Data from the visual acuity test.

We focused on the memory retention values from the three final tests as a function of the spacing interval. No spacing interval preceded the first test of the first learning unit. Results from this first test were thus unaffected by spacing and were taken as individual baselines: For each participant we subtracted these test results from all subsequent test results. The baseline-corrected averages are depicted in Figures 3 and 4.

### Pre-processing of the visual acuity data

During the visual acuity test a Landolt ring was presented repeatedly with 8 possible gap locations. Participants had to indicate the perceived gap location by pressing the appropriate out of 8 possible keys corresponding to the 8 gap locations. If the Landolt size decreases participant's hit rate decreases from 100% to chance level ( = 12.5% for eight possible Landolt gap positions).

The visual acuity values display a logarithmic scaling. Thus, we used the decimal logarithm of the visual acuity as dependent variable of the acuity test in order to get a linear relation between the objective acuity step size constant and the subjective visually experienced step size constant and to allow appropriate inference statistics, e.g. [25], [26].

Subsequently and analogous to the treatment with the vocabulary data we subtracted individually the acuity data of the first test unit from all subsequent visual acuity values (baseline correction).

### Inference Tests

For both the vocabulary experiment and the visual acuity experiment we performed mixed-model ANOVAs with the between subjects factor SPACING (6 steps) and the within subject factor TEST. Only a small number of participants performed the last final test after about 4 weeks (within each spacing condition less than half the subjects). Repeated measures ANOVAs including all three steps of the factor TEST had to be restricted to this smaller number of participants. We calculated a second almost identical ANOVA with only the first and second final test as factor steps and thus a higher number of subjects per spacing condition.

According to our initial hypothesis we expected one or more performance peaks as a function of spacing and retention intervals. This was tested by including polynomal contrast tests in our ANOVAs

## Results

### Vocabulary Experiment

We performed two mixed model ANOVAs with the between-factor SPACING (six steps) and the within-factor TEST. In ANOVA I the factor TEST contained only the first and second final test. In ANOVA II the factor TEST contained all three final tests but with much less data, because only a subset of participants performed the last final test four weeks after the last learning unit.

ANOVA I revealed both a significant effect of the between-factor SPACING (F(5,101) = 4.31, p = .0014) and a significant effect of the within-factor TEST (F(1,101) = 43.72, p = 1.82×10^−9^) reflecting the decay of memory with time. No interaction between factors was found.

ANOVA II showed a smaller non-significant effect size for the between-factor SPACING (F(5,42) = 1.53, p = .2) but still a significant effect for the within-factor TEST (F(2,84) = 59.26, p = 2×10^−16^) and additional an interaction between SPACING and TEST (F(10,84) = 2.05, p = .038).

Table 4 (second column) shows results from the ANOVA polynomal contrast analysis. If only the data from the first and second final test are included, all four fits provide significant p-values with the worst result for quadratic fits. If the data from all three final tests are included, the cubic trend dominates while the 4^th^ order trend does not reach significance.

**Table 4:** Polynomal contrast analysis results

	Vocabulary Experiment	Visual Acuity Experiment
**1^st^ order (linear)**	0.001 (0.029)	0.12 (0.45)
**2^nd^ order (quadratic)**	0.006 (0.012)	1.3•10^−5^ (2.4•10^−7^)
**3^rd^ order (cubic)**	0.002 (0.001)	0.38 (0.08)
**4^th^ order**	0.002 (0.2)	0.03 (0.03)

p-values from the ANOVA polynomal contrast analysis for the factor SPACING. p-values without parantheses: only first and second final tests entered the analysis. p values in parantheses: all final tests entered the analysis.

### Visual Acuity Experiment

Analogous to the vocabulary experiment we performed two ANOVAs for the visual acuity experiment. In ANOVA I the factor TEST has only two steps (first and second final test). In ANOVA II the factor TEST contains all three final tests but has less data, as detailed above.

ANOVA I revealed a significant effect for the factor SPACING (F(5,94) = 3.88, p = .003) but neither effect for the factor TEST nor an interaction between factors. ANOVA II confirmed the result from ANOVA I with a main effect of the factor SPACING (F(5,30) = 4.54, p = .003).

Table 4 (third column) shows results from the ANOVA polynomal contrast analysis. Only the quadratic and the 4^th^ order trend reached significance with a dominance for a quadratic effect.

## Discussion

We studied spacing effects for two different practice domains, vocabulary learning and visual acuity tests, for a broad range of spacing intervals from 7 min to 24 h and three different retention intervals from one day to 28 days.

With the vocabulary experiment we found for all spacing intervals a monotonic increase in memory performance during the learning period (Fig. 3a, blue traces) and a monotonic decrease in memory performance during the test period (Fig. 3a, red traces). The best memory performance at the end of the learning period was observed for the two shortest spacing intervals (7 min and 20 min) with an average of 28 and 27 memorized word pairs, respectively, (i.e. around 68%) out of 40. A spacing interval of 24 h caused the worst performance with about 18 memorized words ( = 46%).

**Figure 5:**
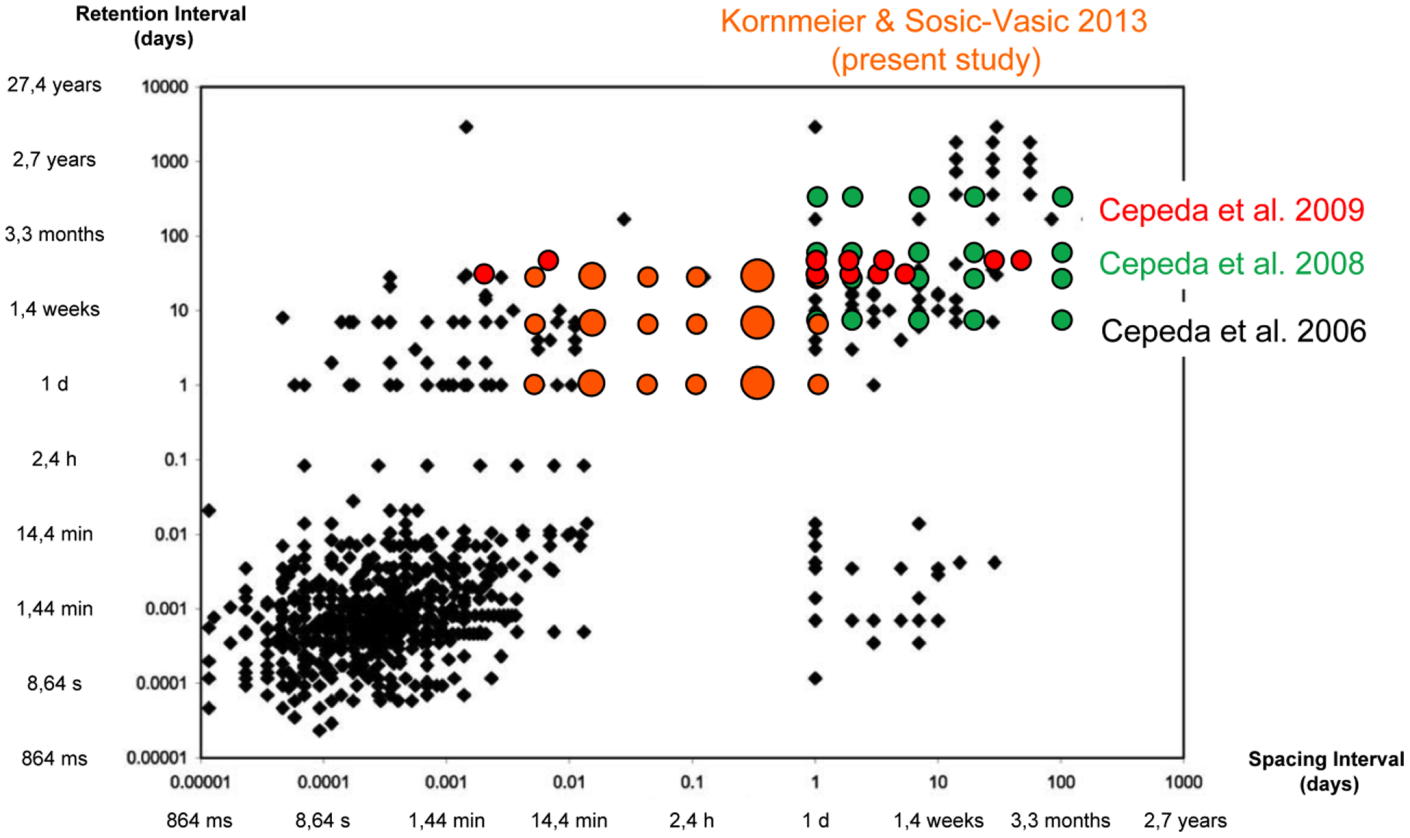
Overview of the spacing intervals (abscissa) and retention intervals (ordinate) used in a number of representative studies. Dots indicate studies with specific spacing/retention interval combinations. Black dots are from an extensive review by Cepeda et al. [2]. Green and red dots are from recent studies of this group. The orange dots indicate spacing and retention intervals used in the present study. The larger orange dots indicate spacing intervals with local and global performance maxima.

Best long-term memory retention was observed for the 12-h-spacing interval with retention of all learned word pairs (100%) after one week and retention of 85% after four weeks. The worst long-term memory retention was again observed with a spacing interval of 24 h with retention of only 33% after four weeks. In the case of the shortest spacing intervals, retention of 78% after one week and 57% after four weeks was observed. Both figures 4 and 5 and our trend tests provided some evidence for a non-linear pattern of the spacing data with a trend order between 2 and 4 and at least one memory performance peak at a spacing interval of about 12 h spacing.

The data from the visual acuity experiment are more variable than those from the vocabulary experiment. Monotonic increase in visual acuity performance during the learning period can only be observed for spacing intervals of 20 min and 12 h. Respectively, monotonic decay of visual acuity test performance is only observable for spacing intervals of 2.5 h and 24 h. And in contrast to the vocabulary experiment, visual acuity test performance in the final test period can be superior to the visual acuity values from all previous learning units (e.g. the spacing conditions of 20 min, 1 h, 2.5 h and 12 h).

Besides these differences there are also remarkable similarities between the two experiments.

First, for both types of tasks the 12-h-spacing interval provides best long term learning performance and the least variability. 85% of the learned word pairs and also a gain in visual acuity of 0.15 log(Visual Acuity) – this can be translated to a gain of 1.5 lines in a typical Visual Acuity Chart – are retained over (at least) four weeks.

Second, for both types of tasks the 24-h-spacing interval provides the worst performance.

Third, there is strong evidence for a nonlinear trend of the factor SPACING with an order between two and four.

Before we interpret our data we have to make some preliminary comments.

### 1. Number of Independent Variables

In the current study we varied spacing and retention interval durations and the learning/practice material (word pairs and visual acuity tests). There are several other independent variables that we had set to certain constants or adaptive values but that may be also highly relevant for learning and memory retention. Among those are (1) the presentation duration of the word pairs and the Landolts, (2) the total number of learning/practice items, (3) the duration of a learning unit (constant time, as used in the current study, or variable time until an a priori defined learning level is achieved), (4) the number of learning units and spacing intervals.

The present findings may be specific for our choice of these variables and the pattern may change with changing them. Differences between the present findings and findings from other studies with similar spacing and retention intervals may be explained by different choices of these independent variables.

### 2. Timing Precision

Studies like the present one are highly demanding. Our participants had to visit the lab up to seven times at certain fixed time points. This is challenging with moderately paid volunteers and the inevitable variability of temporal precision may affect the signal-to-noise ratio of the data and is presented in Tables 3 and 4. Especially the 12-h-spacing condition with the most interesting results was the most problematic one: During the learning period participants had to visit the lab 2–3 times. Strictly maintaining the intended 12-h-spacing would have requested highly flexible participants, which are rare. Due to the expected timing problems, our participants from the 12-h-spacing group had an average spacing interval of 16 h±6 hours. We are thus in the paradoxical situation that the experimental condition with the largest temporal variance is also the condition with the best long-term memory performance and the least memory performance variance. This “optimal” spacing interval of 12 h may thus only represent an order of magnitude and not a precise value.

In a follow up study we plan to increase both temporal precision and temporal duty cycle around spacing intervals of 2.5 h and 24 h to more precisely describe the topology of the SRP-subspace surrounding the global peak in this interval.

We will now discuss our results in the context of previous findings.

#### (1) Massed vs. spaced learning

At the end of the learning period of the vocabulary experiment we find a clear superiority of the short over longer spacing intervals. This picture starts to change already after the first retention interval of one day, when the two short spacing intervals (7 min and 20 min) and the spacing interval of 12 h provide roughly equal results (Fig. 4a). After retention intervals of 1 week and of 4 weeks the 12-h spacing interval clearly dominates all other spacing intervals. The superiority of massed practice for short retention intervals and longer spacing for long retention intervals is one of the core findings of the spacing effect and our results are in good agreement with the previous literature ([2], for reviews,[3]). In the visual acuity experiment we do not observe an initial superiority for short spacing intervals but it is also the 12-h spacing interval that leads to the largest long-term effects in visual acuity test performance.

#### (2) Relation between spacing and retention intervals

A number of studies reported about a nonlinear relation between spacing interval and memory performance with a steep increase and a slow decrease, e.g. [2], [27], [28]. Further, there is some evidence for a positive correlation between the optimal spacing interval duration and the retention interval duration, i.e. the time between the last learning unit and the final test [2], [19], [29], [30].

We also found a nonlinear relation between spacing intervals and memory performance (the slow performance decrease for longer than optimal spacing intervals is hidden in our graphs by the logarithmic scaling) with at least one peak in the SRP-subspace at the spacing interval 12 h (Figs. 4a and b). Figure 4 indicates an additional peak at shorter spacing intervals in the range of minutes. Although our trend analyses do not rule out such a possibility the picture is not clear enough to make strong statements. Our data deviate from the above-mentioned literature in at least one point: Our optimal spacing interval does not increase with the retention interval duration but stays nearly unchanged at a spacing interval of 12 h across retention intervals. Remarkably, this pattern of results can be observed for both the vocabulary and the visual acuity experiment. How can this be explained?

### The Role of Sleep for The Spacing Effect

One obvious feature in our paradigm is that some of the experimental conditions contained sleep phases during the spacing intervals and others not. In the 12-h spacing condition at least one spacing interval contained a sleeping phase and in the 24-h spacing condition each of the spacing intervals contained a sleeping phase. There is plenty of evidence for an important role of sleep for memory consolidation, e.g. [31], [32], [33]. The apparently easiest way to interpret our data may thus be to argue that the maximal memory performance in the 12-h spacing condition may be simply an effect of sleep. The bad performance in the 24-h-condition may then be explained in the following way: Transformation of contents from short to long(er) term memory can only take place with contents from the short term memory at the transformation time. With a spacing interval of 24 h too many items may have been lost from short term memory before sleep-related consolidation starts. One way to test this would be to systematically vary the day times of practice-unit starts. In the current study we were not able to control this variable systematically, but we think this is an important issue for a separate study.

One argument that weakens the simple explanation from above at least partly can be accessed most clearly from the vocabulary data but also from the visual acuity data (Fig. 4 a and b): Long term memory performance is weaker with spacings in the minute range but already increases with a spacing interval of 2.5 h. In the 2.5-h spacing condition, memory performance is very close to the 12-h spacing condition in the final test after one day (FT1) and already rises in the final test after one week (FT2) and after four weeks (FT3). But remarkably, no participant from the 2.5-h spacing condition slept between practice units. Thus a substantial part of the second and global maximum must be due to spacing independent of sleep.

### Some Speculations

We here present a somewhat speculative interpretation that is related to observations of learning mechanisms at the cellular level and that is discussed in more detail elsewhere [20]:

(1) After application of a certain stimulation protocol to neighboring neurons, the synaptic transmission is facilitated, i.e. less presynaptic activity is necessary to induce postsynaptic activation of a neighboring neuron than before (“synaptic facilitation”). This is called long-term potentiation (LTP) and is one of the most often discussed candidate mechanisms for synaptic plasticity, e.g. [34]. The amount of this neuronal learning is typically quantified as the survival time of synaptic facilitation.

(2) There is evidence for a refractory period of potentiated neurons during that no further synaptic facilitation is possible, e.g. [35], [36] probably due to physiological restrictions.

(3) Recent evidence indicates three separate steps from short-term to long-term potentiation, e.g. [35], [37]. Each step is characterized by a specific cascade of intracellular processes, ranging from functional changes, like simple changes in molecule-concentration to structural changes, like gene activation and basic reconstructions of the cell morphology. Each step transforms the memory trace into a more durable state and each step is a necessary precondition for the subsequent step.

(4) Each intracellular modulation step has a certain time constant after which the induced processes reverse if no further stimulation takes place, e.g. [37].

(5) There are several free variables that determine the efficacy of the stimulation protocol, e.g. [38]. Among those parameters is the spacing of stimulation bursts [39], [40], [41].

We here speculate that the observed nonlinearity of the spacing effect reflects refractory and reversal time constants of the underlying neural processes. It may also be reasonable that, like on the cellular level, the way from short-term to long-term memory on the behavioral level takes place in steps, that each step may be characterized by a specific refractory and reversal time constant and that these steps depend on those on the cellular level. The 12-h-peak in our SRP-subspace may reflect such a step and other peaks may be present with earlier spacing intervals.

Our hypothesis allows some testable deductions:

(a) Synaptic plasticity mechanisms seem to be very basic for most if not all types of learning. According to our speculation, this should be also true for the spacing effect. The generality of the spacing effect across very different study parameters, as reported in the literature, provides some between-study support for this speculation. The high similarity between the spacing effect patterns during vocabulary learning and visual acuity practice from the present study provide important within-study evidence. More convincing would be potential findings of similar spacing optima in human behavioral studies and during LTP induction in single cells in animal studies. Such studies have to be done in future.

(b) If optimal spacing intervals reflect succeeding steps during memory formation, a combination of optimal short and longer spacing intervals may be superior to a condition with constant spacing. Several studies already exist where protocols with expanding spacing intervals were compared with constant spacing protocols. The evidence is mixed with some study results showing benefits of expanded spacing and others reporting no or even negative effects. For a review see [29]. Comparing the existing studies with expanding spacing intervals is problematic because of confounding additional factors and narrow within-study ranges of spacing intervals. The effect sizes for expanding and constant spacing studies may strongly depend on the choice of the spacing interval durations and particularly – according to our approach – whether they are close to a peak value in the SRP-Space. Based on these considerations it may well be possible that a schedule with appropriate constant spacing intervals is superior to a schedule with inappropriate expanding spacing intervals and vice versa.

As noted above, past literature indicates that the optimal spacing interval increases with increasing retention intervals. According to our hypothesis longer spacing intervals are coupled with later memory steps that make memory traces more durable. And because the earlier memory steps have to be passed before, we should expect local maxima for optimal shorter and optimal longer spacing intervals when they are combined. Our results may provide a good starting point for a study with expanding spacing schedules combining spacing intervals in the minutes range with spacing intervals around 12 h.

One central question for the most efficient learning schedule concerns the number of peaks in the SRP-space and their locations. Cepeda et al. [2] presented in their impressive review a graphical overview with dots representing representative spacing studies with certain spacing and retention intervals. We present an updated variant of this graph in Fig. 5 where we have added some newer studies. It is obvious that the majority of studies used a narrow range of small spacing and retention values between 1 s and and 1 h. This is reasonable because studies with small spacing and retention values are much easier to realize than studies with long values.

Remarkably, our optimal spacing interval of ±12 h lies in one large white area (no studies so far) within this plane. It will be an enormous effort to fill the residual white areas with dots. A good starting point would be to fill the gaps around the 12 h spacing interval in both directions.

### Summary and Conclusions

In the present study we focused on two aspects of the spacing effect: First, we extended the typically narrow within-study ranges of spacing intervals to values from 7 min to 24 h and of retention intervals to values from 24 h to four weeks and looked for the number of optimal spacing/retention interval pairs. Second we looked for the within-study generality of spacing effects and its optimal spacing values across two very different learning/practice tasks, namely vocabulary learning and visual acuity tests.

The similarity of effects between experiments indicates very basic mechanisms for memory formation with a global maximum at a spacing interval of 12 h across experiments and retention intervals. Remarkably, 12 hours are close to the average sleep duration of a normal adult and several studies demonstrate clear memory effects of sleep, e.g. [31]. Our 12-h spacing effect cannot be explained by consolidation effects during sleep. It may thus be interesting to go the other way round, i.e. to study whether the spacing effect may be a contributing but so far overlooked factor in sleep-dependent memory effects.

The SRP-Space still contains several white areas and we currently do not know about the number of peaks in spacing-dependent memory performance and their distribution. Existing spacing studies differ in a number of relevant parameters, which makes comparisons difficult, e.g. [2], [29]. Studies covering a broad range of spacing and retention intervals, while controlling for other relevant variables, are highly challenging but necessary to get the picture much clearer and to develop reliable advice for application in schools and universities.

Currently it is unclear how learning effects observed on the level of single neurons can be related to learning effects on a behavioral level where probably millions of neurons are involved. But there are already some studies demonstrating such relations between these different levels of complexity, e.g. [42], [43], [44]. Further, there are studies, demonstrating spacing effects on cellular levels, as noted above. Most interesting would be studies where optimal spacing constants at the cellular level could be directly compared to those at the behavioral level. We hope that the present study stimulates research in these directions.
